# Thermal Energy Storage (TES) Prototype Based on Geopolymer Concrete for High-Temperature Applications

**DOI:** 10.3390/ma15207086

**Published:** 2022-10-12

**Authors:** Mohammad Rahjoo, Guido Goracci, Juan J. Gaitero, Pavel Martauz, Esther Rojas, Jorge S. Dolado

**Affiliations:** 1 Centro de Física de Materiales, CSIC-UPV/EHU, Paseo Manuel de Lardizábal 5, 20018 Donostia-San Sebastián, Spain; 2 TECNALIA, Basque Research and Technology Alliance (BRTA), Parque Tecnológico de Bizkaia, Astondo Bidea, Edif. 700, 48160 Derio, Spain; 3 Považská Cementáreň Cement Plant (PCLA), Ulica Janka Kráľa, 01863 Ladce, Slovakia; 4 Plataforma Solar de Almería (PSA-CIEMAT), Av. Complutense 40, 28040 Madrid, Spain; 5 Donostia International Physics Center (DIPC), Paseo Manuel de Lardizabal 4, 20018 Donostia-San Sebastián, Spain

**Keywords:** cement, concrete, geopolymer high-temperature TES, OPC, thermal energy storage

## Abstract

Thermal energy storage (TES) systems are dependent on materials capable of operating at elevated temperatures for their performance and for prevailing as an integral part of industries. High-temperature TES assists in increasing the dispatchability of present power plants as well as increasing the efficiency in heat industry applications. Ordinary Portland cement (OPC)-based concretes are widely used as a sensible TES material in different applications. However, their performance is limited to operation temperatures below 400 °C due to the thermal degradation processes in its structure. In the present work, the performance and heat storage capacity of geopolymer-based concrete (GEO) have been studied experimentally and a comparison was carried out with OPC-based materials. Two thermal scenarios were examined, and results indicate that GEO withstand high running temperatures, higher than 500 °C, revealing higher thermal storage capacity than OPC-based materials. The high thermal energy storage, along with the high thermal diffusion coefficient at high temperatures, makes GEO a potential material that has good competitive properties compared with OPC-based TES. Experiments show the ability of geopolymer-based concrete for thermal energy storage applications, especially in industries that require feasible material for operation at high temperatures.

## 1. Introduction

One of the important factors in intermittent renewable power sources, such as concentrated solar power (CSP) and solar heat for industrial processes (SHIP), and in waste heat recovery industries is optimal dispatch. A power dispatch optimization method in the thermal industry is the implementation of thermal energy storage (TES) systems. In general, TES assists in enhancing foreseeability, capacity, and managing the state of generation, as well as distribution in energy. High-temperature TES applications sometimes face infrastructure constraints as they require apparatus, materials, and heat transfer fluids capable of working at high-temperature regimes, up to ~1000 °C [1].

The stored amount of heat in sensible TES (*Q_S_* (J)) is proportional to the mass of storage material (*m* (kg)), its heat capacity (*C_p_* (J/kg °C)), and the difference between the storage material final and initial temperature (Δ*T* (°C)), Equation (1). Therefore, the maximum achievable thermal storage capacity is vastly reliant on achievable Δ*T* [2].

(1) $$Q_{s}=m\cdot C_{p}\cdot \Delta T$$

Even though TES can have different forms and methods [2], a sensible type of TES has touched the necessary level of maturity in CSP and SHIP and started to establish markets by employing molten nitrates as a storage medium. Moreover, despite the fact that molten salts have an attractive cost (20–50 c€/hWeh; [3]), solid-state materials, such as ceramics, rocks, and concretes are considered as the most attractive approaches for decreasing the capital and expenditure costs of TES systems, as well as increased lifespan and easier maintenance. Implementing solid-state TES materials does not require pressure vessels and transfer systems, as well as heat tracing systems that are typically the main constraints of using molten salts.

Concrete, a composite formed upon mixing cement binders, aggregates, admixtures, and water, is one of the most abundant and promising candidates among solid-state materials [2]. Concrete is low-cost, durable, and requires almost no maintenance [2]. Ordinary Portland cement (OPC) concrete is the most investigated solid-state TES for solar thermal applications [4,5,6,7,8,9,10,11,12]. Lating et al. [4] introduced an OPC-based concrete (DLR) capable of operation between 200 and 400 °C. Skinner et al. [5] tested a lab scale OPC-based concrete (UHPC) at temperatures between 400 and 500 °C. Similarly, Hoivik et al. [6] investigated and tested an improved OPC-based concrete (Heatcrete) capable of working at temperatures of up to 380 °C. The main drawback that limits the application of OPC-based concrete is working temperature limitation. Thermal properties of OPC-based composites are controlled by C-S-H gel (in Cement Chemist Notation C = CaO, S = SiO_2_, H = H_2_O), which is the key product resulting from hydration processes in OPC binders. Nevertheless, the C-S-H gel degrades at temperatures between ~400 and 600 °C [13,14].

There are other families of cements apart from OPCs. Calcium sulfoaluminate (C$A) cements are currently proposed as promising TES media [11,15]. These cements produce ettringite (Ca₆Al₂(SO₄)₃(OH)₁₂·26H₂O) as the main reaction product. Its high-water content and rich hydrogen network [16] make Ettringite very attractive for thermochemical energy storage purposes. Other cements, such as belite (BCs) [17,18], calcium aluminate (CA) [19], and geopolymers (GEO) [20,21,22] are known to withstand higher temperatures than OPCs. GEO composites have attractive properties. They are produced from secondary products [23] and their CO_2_ fingerprint is lower than OPC. Studies and experiments have shown that GEOs have better thermal stability at elevated temperatures [24,25,26,27]. In addition, we have recently reviewed and concluded that geopolymers have a higher content of nanoconfined water in their main hydration product, the N-A-S-H (N = Na_2_O, A = Al_2_O_3_), which is a hallmark for an improved and stable heat capacity [28].

This work presents the results of experiments performed in a pilot thermal energy storage test facility with hot air as inlet heat transfer fluid. In essence, this work is a continuation of the previous work, which was a proof-of-concept for GEO-based thermal energy storage devices [28]. Thermal energy storage capacity and thermal behavior of GEO- and OPC-based TES modules were studied in two thermal scenarios, and study temperatures were 650 and 450 °C. Moreover, the thermal cyclic behavior for charging and discharging TES modules was assessed. In the end, the amount of thermal energy storage obtained in different scenarios was investigated and compared. Furthermore, a comparison with a state-of-the-art sample [6] was carried out and the results were presented and compared.

## 2. Materials and Methods

### 2.1. Sample Preparation

The industrially produced geopolymer hybrid cement from Považská Cementáreň [29] was used as a binder to create the GEO concrete module, employing w/c = 0.6 and 75% wt of steel slag aggregate. The geopolymer hybrid cement contains 20% of Portland cement clinker and 80% of inorganic geopolymer. In addition, it assists in maintaining a stable and high heat capacity over different temperature ranges, while slag maintains a proper thermal conductivity. Moreover, a sample of OPC concrete whose properties and formulation are presented in Table 1 was prepared. Cubes of 10 cm^3^ were prepared to check the quality of samples after 90 days. Then, the compressive strength of samples was measured as 47.25 and 50.87 MPa for GEO and OPC, respectively.

### 2.2. Module Description

**Figure 1:**
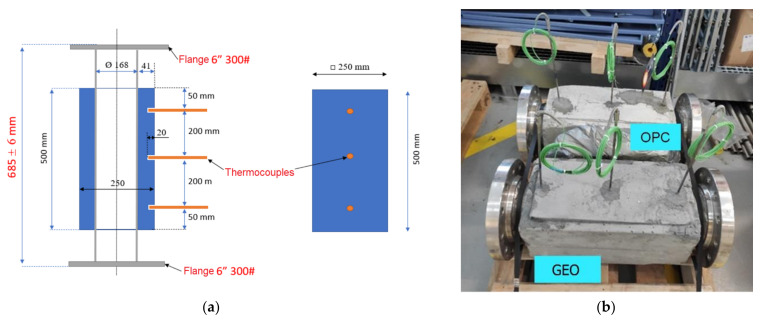
(**a**) Scheme and dimensions of the TES prototypes; (**b**) photograph of the concrete TES modules.

For sample preparation of thermal energy storage devices, the Embedded tube scheme is inspired, Figure 1. A schedule of 40 stainless pipe 304/304L Welded tubes of 6″ (inch) was used as the heat exchanging pass for heat transfer fluid (HTF). Moreover, two flanges were installed at both ends of the tubes to connect the TES sample on the test facility. Three temperature probes were embedded 20 mm in the concrete to measure and read the temperature of the TES modules. The experiment was carried out in the CIC EnergiGUNE, Álava, Spain.

### 2.3. Thermal Cycles Setup

**Figure 2:**
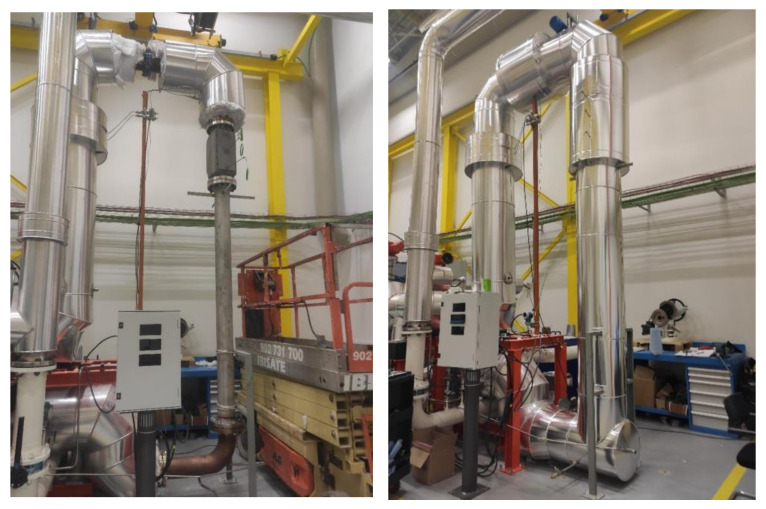
Thermal energy storage testing loop.

For the TES experiment setup, air was chosen as the circulating heat transfer fluid (HTF). The HTF is heated by an electric heater and then supplied to the TES modules by a pump, Figure 2. The TES modules are flanged onto predetermined air heater tubes, followed by the insulation and jacketing process for the highest possible sealing. Better insulation will be effective in reducing thermal losses during the experiment. For evaluating the performance of the prototypes, two running scenarios were studied. First, a low-temperature scenario, with four cycles of charges and discharges and running temperatures of the inlet air temperature (T_inlet_) between 200 and 450 °C that correspond to the typical conditions reported for OPC-based concrete (Heatcrete) [6]. Then, a high-temperature scenario, with four cycles of charges and discharges and T_inlet_ varying between 200 to 600 °C, which clearly overpasses the temperature threshold of the state-of-the-art OPC sample. The goal is to observe the GEO workability in the higher temperature range. Real-time measurements were carried out to monitor the operational performance of different TES modules. For this purpose, the temperature and velocity of the incoming air, as well as the temperature of the samples at three specified intervals, were recorded by a thermocouple, as shown in Figure 1.

## 3. Results

**Figure 3:**
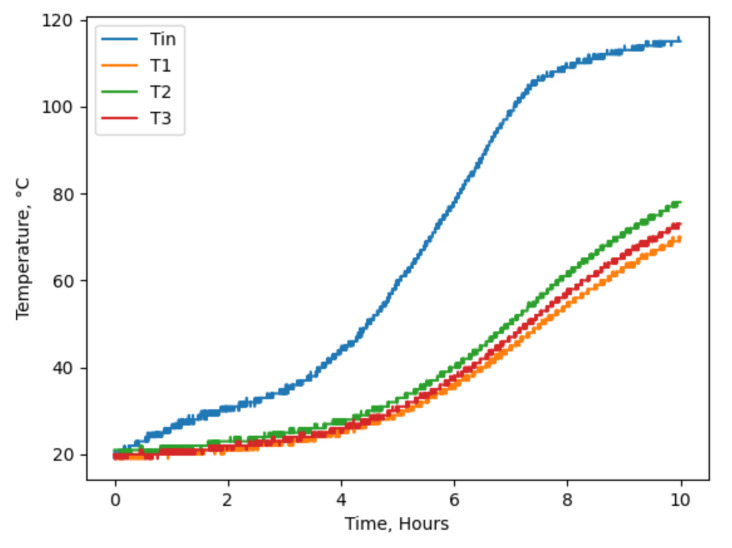
Startup scenario and first heating ramp for the first 10 h, GEO sample. T_1_,T_2_, and T_3_ are thermocouples shown in Figure 1. T_in_ is the HTF (air) inlet temperature.

To conduct the experiment, four thermal cycles were considered, with each complete cycle lasting approximately 24 h. The aim was to investigate the behavior of samples for thermal energy storage analysis. In this regard, two thermal scenarios, T_inlet_ = 450 °C and T_inlet_ = 650 °C, were considered. To start the experiment and prevent thermal shock, a startup scenario was considered. Air flow temperature (T_inlet_) was slowly increased over time by up to about ~120 °C within 10 h, Figure 3. Temperature values of T_1-3_ show the temperature variation along the flow axis, and temperature probes are shown in Figure 1b. The considered preheating rate before running the experiment was 3 °C/h for T_inlet_ = 450 °C and 14 °C/h for T_inlet_ = 650 °C. The latter has a higher heating rate as the sample was already subjected to thermal conditions. 

**Figure 4:**
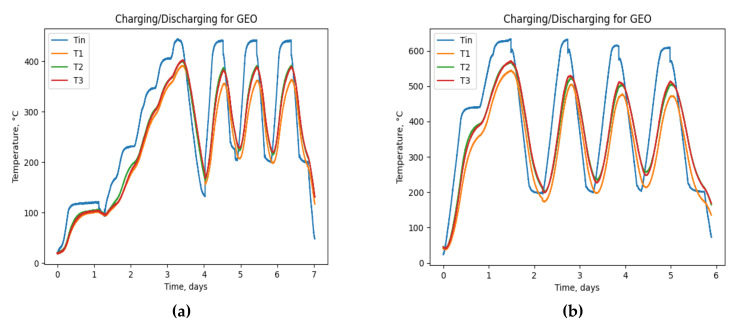
GEO concrete. (**a**) Low-temperature cycles (the temperature of the inlet varying from 200 ± 25 °C to 400 ± 25 °C); (**b**) high-temperature cycles (the temperature of the inlet varying from 200 ± 25 °C to 600 ± 25 °C). T1,T2, and T3 are thermocouples shown in Figure 1. Tin is the HTF (air) inlet temperature.

**Figure 5:**
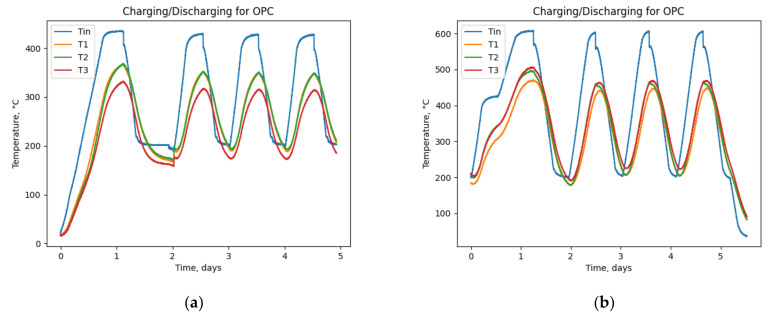
OPC concrete. (**a**) Low-temperature cycles (the temperature of the inlet varying from 200 ± 25 °C to 400 ± 25 °C); (**b**) high-temperature cycles (the temperature of the inlet varying from 200 ± 25 °C to 600 ± 25 °C). T1,T2, and T3 are thermocouples shown in Figure 1. Tin is the HTF (air) inlet temperature.

The maximum average temperature obtained from GEO and OPC samples during the experiment in both low (T_inlet_ = 450 °C) and high (T_inlet_ = 650 °C) scenarios are shown in Table 2. Moreover, the full experiment data for high and low conditions on two samples are shown in Figure 4 and Figure 5. Considering the third and fourth cycles as more thermally stable conditions, the temperature difference between GEO and OPC reveals that in T_inlet_ = 450 °C, the maximum temperature difference obtained for the GEO sample is ΔT = 30 °C higher than OPC, and for T_inlet_ = 650 °C the difference is ΔT = 40 °C. Although it is expected that the GEO sample will perform well in high-temperature scenarios, the results indicate the acceptability and good competitiveness of the GEO sample in T_inlet_ = 450 °C. This work and its assessment with the previous study at the lab level [28] agree that the GEO concrete excels at high-temperature regime (>400 °C), where thermal diffusivity remains stable.

**Figure 6:**
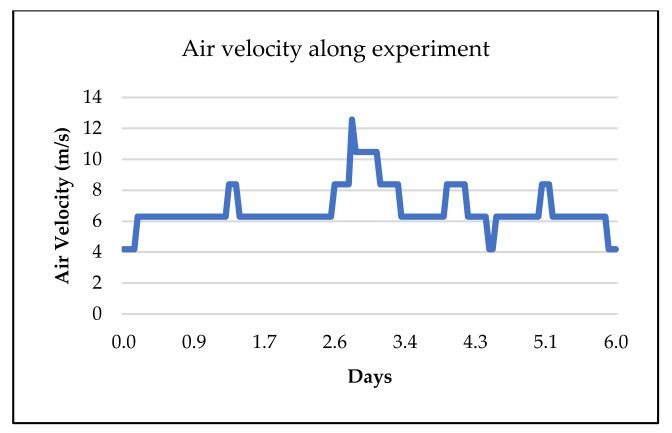
Measured heat transfer fluid (HTF) velocity during continuous cycling for TES modules over a period of 6 days.

An average speed of 6 m/s was considered for incoming heat transfer fluid (HTF). The comprehensive pattern of HTF speed variation during the experiment is shown in Figure 6. Moreover, the average flow rate for HTF was ±412 m^3^/h and the maximum and minimum values were 825 and 275 m^3^/h, respectively.

## 4. Discussion

According to the results and graphs obtained from the previous section, Figure 5 and Figure 6, other characteristics of a TES device, such as thermal energy storage capacity (Q) can be estimated, using Equation (1). For this purpose, it is necessary to have the thermal properties of the samples available. Of note, the thermal properties of the tested OPC sample are unknown. The values from the state-of-the-art OPC sample [6] have been used for evaluation and comparison purposes with the GEO data, as shown in Table 3.

The amount of stored thermal energy for the charge cycles of the GEO sample are compiled for two scenarios of low-temperature (T_inlet_ = 450 °C) and high-temperature (T_inlet_ = 650 °C) in Table 4. Since this table briefly refers to the operable temperature range and difference, and at the same time the storage capacity of thermal energy on a volumetric scale (Q_vol_), it is used as the main criterion for ensuring the thermal performance of TES samples.

The average temperature of OPC-based concrete (Heatcrete) [6] in its working cycles considering only the charging cycles are extracted. Moreover, with this information, the volumetric thermal energy storage capacity (Q_vol_, MJ/m^3^) was calculated, as shown in Appendix A and in Table A1. Considering that the thermal properties of the OPC sample are more competitive than GEO, the limitations of the operating temperature of the OPC lead to the fact that an obtainable ΔT cannot exceed a certain range, and this leads the Q_vol_ (computable by Equation (1)) to always remain in a specified range of about 260–265 MJ/m^3^. Considering that the GEO sample can be utilized in a wider temperature range, naturally, a higher ΔT and higher Q_vol_ will be obtained. This value for the GEO sample varies between 465–942 MJ/m^3^, which in its lowest case is two times higher and up to 3.5 times higher than the OPC sample. In both low- and high-temperature scenarios of the GEO sample, we consider only 2, 3, and 4 as the standard working cycles for thermal storage temperature range and for averaging the volumetric thermal storage amount (Q_vol_). The average Q_vol_ can be compared with the Heatcrete sample, and the results are displayed in Table 5.

As can be seen in Table 5, the GEO sample has a higher thermal storage capacity due to the ability to work in a wider temperature range. This feature, along with the ability to work at higher temperatures, makes the GEO material applicable in more applications, especially in solar energy-related industries to fill the gap between generation and storage capacity, since these industries demand materials that can work in broader and higher temperature range [1]. Moreover, the need for durable, economic, and high-temperature TES is felt in applications, such as converting electricity to thermal energy, and recycling excess heat in industries. In the next stages of development of this research, which will include upscaling and industrial prototypes testing under real solar facilities conditions, it is essential to have a strong simulation model that can well predict the performance of the TES device. Moreover, having a robust numerical model will play an effective role in the design of the heat exchanger as it is directly related to the quality of charge/discharge.

## 5. Conclusions

In this work, the geopolymer-based concrete (GEO) was analyzed and investigated as an alternative to OPC-based concrete for solid-state high-temperature TES. GEO concrete can work in a wider temperature range, which leads to an increase in thermal energy storage capacity and, as a result, an increase in TES total performance. In particular, experimental measurements show that under similar laboratory conditions, the GEO concrete withstands high running temperatures, higher than 500 °C, than the OPC-based counterpart. Moreover, numerical results and simulations show that the thermal energy storage capacity and temperature difference (ΔT) in charge and discharge cycles are within a very acceptable range as a TES material. The thermal storage capacity of GEO concrete can be higher by up to 3.5 times than the OPC-based state-of-the-art concrete. Correspondingly, GEO utilization as TES may lead the solar thermal generation capacity and TES capacity to be somewhat closer to each other and resolve the mismatch between power generation and demand. Considered together, this work proves that GEO concretes are among the capable choices in order that CSP, SHIP, and waste heat recovery industries can operate and store thermal energy at higher temperatures with geopolymer-based materials. Ultimately, of course, future steps should include the design of industrial scale GEO TES modules to monitor the material’s performance and heat exchanger efficiency under real operating conditions. Therefore, the first step to scale up and for the industrialization of GEO-based TES is to have a valid computational model based on the carried out experiment. A robust computational fluid dynamics (CFD) mechanism assists in predicting the thermal behavior of GEO and modeling the TES module under different conditions and scales.

## Figures and Tables

**Table 1:** Formulation of the GEO and OPC concretes.

Material	GEO	OPC
Cement	9.90 [kg]	9.00 [kg]
Limestone aggregates 0/12 ^1^	-	35.25 [kg]
Limestone aggregates 4/12 ^2^	-	24.75 [kg]
SLK aggregates 0/8 ^3^	66.00 [kg]	-
Water	5.78 [kg]	5.25 [kg]
Viscocrete	70.80 [g]	28.00 [g]
Sikament	104.50 [g]	95.00 [g]

Particle size of aggregates: ^1^ 0/12 = 0 to 12 mm; ^2^ 4/12 = 4 to 12 mm; ^3^ 0/8 = 0 to 8 mm.

**Table 2:** Maximum achieved temperature measured from thermocouple 2 for GEO and OPC.

TES Sample	GEO (°C)	OPC (°C)
Inlet = 450 °C	397.7	366
	358	352
	380	350
	380	349
Inlet = 650 °C	561	500
	518	457
	503	463
	503	463

**Table 3:** Thermal properties of samples.

Properties		GEO [28]	Heatcrete ^1^ [6]
Density	kg/m^3^	2890.0	2253
Thermal conductivity	W/m °K	1.2	1.78
Heat capacity	J/kg °K	1000	1280
Thermal diffusivity	×10^−7^ m^2^/s	5.58	6.01

^1^ Data obtained at 400 °C for Heatcrete concrete.

**Table 4:** Summary of data obtained during experiments (GEO).

Scenario	Cycle	Time, h	Tmin, °C	Tmax, °C	ΔT, °C	Q_vol_, MJ/m^3^
T_inlet_ 450 °C	1	81.6	20	397.7	377.7	1091.6
	2	14.9	163	358	195	563.6
	3	12.0	219	380	161	465.3
	4	12.2	211	380	169	488.4
T_inlet_ 650 °C	1	35.3	44	561	517	1494.1
	2	14.9	192	518	326	942.1
	3	14.9	219	503	284	820.8
	4	13.2	240	503	263	760.1

Tmin and Tmax are average values of thermocouples for the GEO sample, as shown in Figure 1.

**Table 5:** Comparison of Q_vol_ for GEO and Heatcrete [6].

Scenario	Cycles	Average Q_vol_, MJ/m^3^
GEO (T_inlet_ 450 °C)	2–4	505.27
GEO (T_inlet_ 650 °C)	2–4	841
Heatcrete (T_inlet_ 400 °C) [6]	1–4	261.25

## Data Availability

Not applicable.

## Appendix A

The average temperature of Heatcrete concrete [6] considering charging cycles are obtained from “Measured internal concrete temperatures, Figure 15” of the aforementioned paper. Then, the volumetric thermal energy storage capacity (Qvol, MJ/m^3^) was calculated.

**Table A1:** Summary of data obtained from Heatcrete [6].

Cycle	Time, h	Tmin, °C	Tmax, °C	ΔT, °C	Q_vol_, MJ/m^3^
1	7.5	260	350	90	260
2	9	262	352	90	260
3	9	265	357	92	265
4	9	270	360	90	260
